# Non-native speech recognition sentences: A new materials set for non-native speech perception research

**DOI:** 10.3758/s13428-019-01251-z

**Published:** 2019-04-22

**Authors:** Louise Stringer, Paul Iverson

**Affiliations:** 1 Department of Speech, Hearing and Phonetic Sciences, University College London, London, UK; 2 Academic Support Office, University of York, York, UK

**Keywords:** L2 speech perception, Stimulus set

## Abstract

Research into non-native (L2) speech perception has increased the need for specialized experimental materials. The Non-Native Speech Recognition (NNSR) sentences are a new large-scale set of speech recognition materials for research with L2 speakers of English at CEFR level B1 (North, Ortega, & Sheehan, 2010) and above. The set comprises 439 triplets of sentences in three related conditions: semantically predictable, neutral, and anomalous. The sentences were created by combining a strongly or weakly contextually constrained sentence frame with a congruent or anomalous final keyword, and they were matched on a number of factors during development, to maintain consistency across conditions. This article describes the development process of the NNSR sentences, along with results of speech-in-noise intelligibility testing for L2 and native English speakers. Suggestions for the sentences’ application in a range of investigations and experimental designs are also discussed.

A move from ideal laboratory conditions toward more realistic communication in speech research and increased global mobility have led to a large increase in research into non-native (L2) speech perception over recent decades. To explore L2 speech acquisition, some of this research has focused on L2-specific factors such as the influence of perceptual training on the identification of L2 phonemes (e.g., Iverson & Evans, 2009; Shinohara & Iverson, 2013; Ylinen et al., 2010) and their accurate production (e.g., Bradlow, Pisoni, Akahane-Yamada, & Tohkura, 1997; Iverson, Pinet, & Evans, 2012). L2 speech perception has also been compared to native (L1) processing, with the key finding being that L2 speakers are disproportionately affected by adverse listening conditions; background noise has a greater impact on speech intelligibility for L2 than for L1 listeners, even at high levels of L2 proficiency, at which listeners might perform similarly to L1 listeners in quiet (e.g., Cooke, García Lecumberri, & Barker, 2008; Crandell & Smaldino, 1996; Mayo, Florentine, & Buus, 1997; Pinet, Iverson, & Huckvale, 2011), and L2 listeners are also affected more negatively by reverberation (Nábělek & Donahue, 1984; Shi, 2014; Takata & Nábělek, 1990). Non-native listeners may also benefit less from cues that aid L1 speech recognition in adverse conditions, such as the availability of contextual clues (Bradlow & Alexander, 2007; Mayo et al., 1997; Shi, 2012) or clear speech enhancements (Bradlow & Alexander, 2007). Further exploration of quantitative and qualitative differences in L1 and L2 speech recognition processes could help identify processes that are general to all speech processing versus processes that are language-specific or arise only in very difficult listening situations.

In spite of this greater focus on L2 speech perception research, few experimental materials have been designed specifically for this purpose, meaning that L2 speech research often relies on materials created for native speakers. A widely used material set is the Bamford–Kowal–Bench (BKB) sentences (Bench, Kowal, & Bamford, 1979), which were developed to assess the speech perception abilities of hearing-impaired English-speaking children. The set comprises syntactically and lexically simple sentences that each contain three or four keywords. Another commonly used set of materials is the Speech Perception in Noise (SPIN) sentences, developed to evaluate the speech perception of hearing-impaired adult English speakers (Kalikow, Stevens, & Elliott, 1977). The SPIN sentences have the advantage of allowing the manipulation of the level of contextual information available, but they contain some very-low-frequency lexical items. Using these materials can be problematic, because their vocabulary and syntax may be linguistically complex (in the case of materials developed for L1 adults) or overly simple (in the case of materials aimed at children). This causes difficulties in ensuring that tasks measure speech perception rather than linguistic knowledge.

To address these issues, some L2-specific experimental materials have been developed. The largest-scale of these sets is the Basic English Lexicon (BEL) sentences (Calandruccio & Smiljanic, 2012), which are based on a lexicon developed from recordings of spontaneous non-native speech. The set consists of 500 sentences, each containing four keywords, and their suitability for administration to L2 English speakers has been demonstrated through large-scale listening tests (Rimikis, Smiljanic, & Calandruccio, 2013). However, the BEL sentences are similar to the BKBs, in that the level of semantic context in the sentences cannot be manipulated, which may limit their use in some experimental designs. Bradlow and Alexander (2007) developed an L2-specific material set that is similar to the SPIN sentences, to allow such manipulation. However, the set contains only 60 pairs of high- and low-predictability sentences, restricting its use to experimental designs with only a small number of within-subjects conditions. In addition, the low-predictability sentences are based on a limited variety of extremely simple sentence structures, so the high- and low-predictability sentences differ in complexity and length in addition to the level of semantic context provided. A more in-depth description of the materials commonly used in L2 speech research is given by Calandruccio and Smiljanic in their presentation of the BEL sentences.

The need for a wider range of L2-specific speech perception materials inspired the development of a new material set, the Non-Native Speech Recognition (NNSR) sentences. The design of the NNSR sentences was informed by three criteria, which were based on the limitations of the materials described above: Lexis and syntax should be appropriate for intermediate-level adult L2 English speakers without being overly simplistic, the set should be of a large scale, and it must be possible to manipulate the level of contextual information provided in the sentences while minimizing other syntactic and lexical differences.

To meet the first criterion, lexical items and syntactic structures were drawn from English language-learning materials designed for speakers at the B1 level of the Common European Framework of Reference for Languages (CEFR). This is an “intermediate” level, at which speakers can successfully communicate on a range of topics but have some gaps in lexical and syntactic knowledge (North, Ortega, & Sheehan, 2010). The second criterion was met by creating matched triplets of sentences in three semantic conditions: predictable, neutral, and anomalous. The semantic conditions are formed from different combinations of a sentence frame (a sentence missing the final word) with either a strongly or weakly constrained semantic context and a final keyword that is either congruous or incongruous to this context (Table 1 shows how each condition is constructed). The corresponding highly or weakly constrained sentence frames and congruous or incongruous final keywords are balanced to minimize the influence of factors such as sentence complexity or word frequency on sentence intelligibility. The complete NNSR sentence set comprises 439 of these semantically related sentence triplets (i.e., 1,317 sentences in total), thus meeting the criterion of creating a large-scale material set. The following sections discuss in more detail the process of developing and validating the NNSR sentences.

**Table 1:** Composition of the three semantic conditions

Condition	Sentence Frame	Final Keyword	Example 1	Example 2
Predictable	Strongly constrained	Congruous	**My shoes are made of brown LEATHER**	**Children like pasta with tomato SAUCE**
Neutral	Weakly constrained	Congruous	My coat is made of nice **LEATHER**	Children like burgers with delicious **SAUCE**
Anomalous	Strongly constrained	Incongruous	**My shoes are made of brown** HOCKEY	**Children like pasta with tomato** NOON

Content overlapping across sentences within a triplet is shown in bold, the pointer words that generate the context are underlined, and final keywords are capitalized

## The development process

To ensure that the NNSR sentences are linguistically accessible to intermediate-level L2 English speakers, the lexical items and syntactic structures used were limited to those found in B1-level English language-learning materials. The words appearing in the NNSR sentences were drawn from the vocabulary list of the Preliminary English Test (PET), which is a B1-level certification (University of Cambridge ESOL Examinations, 2012), and the syntactic structures were restricted to those identified as appropriate for B1-level speakers in the CEFR Core Inventory (North et al., 2010). Although the NNSR sentences are appropriate for intermediate-level L2 English speakers, they will not be overly lexically or syntactically restricted, since the PET vocabulary list contains approximately 3,300 words (University of Cambridge ESOL Examinations, 2012), and the majority of common syntactic structures appear in the B1-level CEFR specifications (North et al., 2010, pp. 10–11).

The development of the NNSR started by identifying a pool of potential final keywords, which were then used to create a large set of semantically predictable sentences. The other conditions were based on these predictable sentences; semantically neutral sentences were created by modifying the sentence frame, whereas semantically anomalous sentences were created by substituting another from the pool of potential keywords for the final keyword.

The initial selection of potential final keywords was limited to nouns, in order to maintain similarity across sentences. To minimize confusion, some nouns were excluded: nouns that are also verbs (e.g., *book*), multiword nouns (e.g., *weather forecast*, although one-word compounds were permitted), hyphenated words (e.g., *make-up*), acronyms (e.g., *DVD*), words with common abbreviations (e.g., *bicycle/bike*), nouns with male/female forms (e.g., *actor/actress*), titles (e.g., *Mr*., *Miss*), and nouns that do not appear in the word property databases used at later stages in development. This left a pool of 1,413 potential final keywords that were between one and five syllables long and had a frequency from 0.04 to 5,250 occurrences per million words (mean frequency = 61 occurrences/million words; Brysbaert & New, 2009). Since the words were drawn from B1-level materials, even the least frequent words were likely to be familiar to non-native participants (e.g., *notepaper*, *footballer*).

Each sentence frame has a context generated by two or three “pointer words.” These are the words that carry the main semantic meaning in the sentence, and are mostly nouns, verbs, or adjectives. For the strongly constrained context, used in both the predictable and anomalous conditions, the pointer words are closely related and generate a specific context (“You wear shoes on your . . .”), whereas for the weakly constrained context, used in the neutral condition, the pointer words are less related and do not generate a specific context (“You have dirt on your . . .”). The predictable and neutral sentences are completed with a congruent (“feet”) final keyword, and the anomalous sentences are completed by an incongruent (“trucks”) final keyword. In line with the existing materials, the sentence frame length was limited to 5–9 words/5–12 syllables (e.g., Block & Baldwin, 2010; Bradlow & Alexander, 2007; Calandruccio & Smiljanic, 2012), giving complete sentence lengths of 6–10 words and 6–16 syllables. Examples of the construction of the three semantic conditions are given in Table 1.

### Semantically predictable sentences

Semantically predictable sentences were created by constructing a highly constrained sentence frame that is predictably completed by a final keyword drawn from the pool. To achieve a high level of predictability for the final keyword, each sentence frame contains two or three pointer words that are closely related to the final keyword, generating a specific context that is likely to be completed by this keyword. For example, a predictable sentence could be formed by combining the highly constrained sentence frame “I drink coffee with cream and . . .” with the final keyword “sugar.” To maximize the size of the final material set, highly constrained sentence frames were created for as many potential keywords as possible, giving an initial set of 553 predictable sentences.

To ensure that final keywords were sufficiently predictable, a series of cloze tests were conducted. In a cloze test, participants receive a list of sentence frames and supply a word to complete the sentence (e.g., “I drink coffee with cream and _______”). No possible options are provided. A word’s cloze probability is the proportion of participants who choose that word to complete the sentence. For example, if eight out of ten participants choose “sugar” to complete the sentence above, it has a cloze probability of 80%. Highly constrained contexts should exhibit high cloze probabilities, showing that the final keyword is predictable (Kutas & Hillyard, 1984). The existing stimulus sets have defined sentences with a cloze probability of around 65% or higher as highly predictable (Block & Baldwin, 2010; Bradlow & Alexander, 2007), so 65% was set as the lower cloze probability threshold for inclusion in the semantically predictable condition. Since the NNSR sentences are intended for use in L2 speech perception research, both native and non-native speakers participated in the cloze tests. To avoid bias toward speakers of any particular language, the L1 of non-native participants was not restricted, and speakers of 21 different L1s took part across the series of cloze tests. The native languages of participants across all cloze tests are given in Table 2.

**Table 2:** Native languages of cloze test participants

Cloze Test P1	Cloze Test P2	Cloze Test P3	Cloze Test N1	Cloze Test N2
*N* & proficient NN	*N* & proficient NN	intermediate NN	*N* & proficient NN	*N* & proficient NN
Albanian (1) Arabic (2) Cantonese (2) Dutch (2) English (18) French (1) German (7) Hungarian (3) Italian (1) Korean (1) Polish (1) Romanian (2) Serbian (1) Spanish (2)	Dutch (1) English (10) French (1) German (2) Romanian (1) Serbian (1) Spanish (2)	Bosnian (1) French (1) German (1) Hindi (2) Hungarian (5) Italian (2) Korean (3) Russian (2) Spanish (9) Thai (1) Vietnamese (8)	English (9) Kiswahili (1) Korean (2) Mandarin (1) Slovak (1)	Cantonese (1) English (9) German (1) Romanian (1) Slovak (1)

The number of speakers of each language is given in parentheses

In Cloze Test P1, the initial 553 predictable sentences were randomly divided into four lists of approximately 140 sentences, which did not differ on the basis of syllable, pointer word, or total word count*.* The cloze tests for each list were completed online by 18 native (13 female, five male) and 26 proficient nonnative (13 female, 13 male; average age of acquisition [AoA] = 9.45 years) English speakers, with a mean age of 29.75 years. The proficient non-native English speakers were all highly fluent in English; all worked or were postgraduate students in an English-speaking environment and used English socially, many lived in an English-speaking country, and none were actively studying English. This was also the case for the proficient nonnative participants in cloze tests P2, N1, and N2. Participants each completed only one test list, were requested to work alone without a dictionary, and were not compensated for their time. The average cloze probability for all sentences across cloze test P1 was 81.8%, showing that final keywords were highly predictable overall (native only, 85.5%; non-native only, 79.0%). The sentences with an overall keyword cloze probability over the 65% threshold were retained unmodified (387), were adapted slightly on the basis of the responses to further strengthen their contextual constraint (54), or were discarded for being too UK-centric (7). One sentence was found to have 100% cloze probability, but the given response was not the intended keyword. However, this alternative response also appeared on the potential-keyword list, so it was substituted for the initial keyword in the retained sentence. The sentences falling under the 65% cloze probability threshold were either adapted in order to reduce their contextual ambiguity (62) or discarded (42).

Cloze test P2 was then carried out to assess whether the 116 sentences modified after cloze test P1 now reached the 65% cloze probability threshold. A new cloze test was completed online by ten native (seven female, three male) and eight proficient nonnative (four female, four male; average AoA = 9.88 years) English speakers, with a mean age of 31.4 years. Again, participants worked alone without a dictionary and were not compensated for their time. The overall cloze probability for these sentences was 79.0% (native only, 80.3%; non-native only, 77.2%). The sentences that now reached the 65% threshold were retained (88), with one sentence being adapted very slightly on the basis of the responses to reduce its ambiguity. One sentence that had reached the 65% threshold in cloze test P1 but that had been modified in an attempt to further strengthen the semantic context was returned to its original form, since the modified sentence was found to have a lower cloze probability rating. Sentences under the 65% threshold were either discarded (23) or adapted on the basis of the responses, if their cloze probability was very close to 65% (3). Along with the 388 sentences retained after cloze test P1, this gave a set of 481 sentences with an average cloze probability of 90.2% (native only, 91.7%; nonnative only, 88.2%). This set of 481 semantically predictable sentences was then used to develop the semantically neutral and anomalous sentences.

Cloze test P3 was conducted in parallel with the subsequent stages of development, to ensure that these predictable sentences are also suitable for intermediate-level English speakers. This is necessary, since these listeners might be less able to take advantage of the semantic cues provided by pointer words than the native and proficient non-native participants in cloze tests P1 and P2. The 481 predictable sentences remaining after cloze test P2 were divided into four lists of approximately 120 sentences, which did not differ on the basis of the numbers of pointer words or syllables or of total word count. A cloze test was compiled for each list and completed either in pen-and-paper form or online by 36 participants (19 female, 17 male; mean age = 27.7 years, mean AoA = 7.5 years). All participants were English language students currently enrolled in preintermediate (1), intermediate (17), or upper-intermediate (18) classes, which equate to CEFR levels A2, B1, and B2. The participants completed one cloze test, were asked to work alone without a dictionary, and were not compensated for their time. One upper-intermediate-level participant’s responses were excluded because that participant completed only a small part of the test.

The average cloze probability of the final keywords in cloze test P3 was 67.9%, showing that the sentences were less predictable for English language learners than for the native and proficient L2 participants in the previous cloze tests. However, the majority of the sentences did exceed the 65% cloze probability threshold for the intended keyword (284). For sentences that did not meet the threshold, the responses given were often closely related to the intended keyword. For example, to complete “Your hands are connected to your _______,” the intended keyword “arms” had a cloze probability of 50% (as compared to 89% for the L1 and proficient L2 participants in cloze test 2), with “body” and “shoulders” also being given in response. There were also a small number of sentences with a cloze probability over 65% but for which the most common response was semantically related to, but not, the intended keyword (7). These semantically related responses suggest that in most cases the relevant context was activated by the sentence frame, and participants were able to anticipate the final keyword, even though they might ultimately select a different word from the intended keyword. However, some sentences did not seem to be predictable for these lower-level participants, so these sentences were either discarded (9) or modified for clarification (5). The corresponding neutral or anomalous sentences were also removed or adapted accordingly.

### Semantically neutral sentences

A semantically neutral counterpart was created for each predictable sentence by substituting for the pointer words in the strongly constrained sentence frame words that are less closely related to the final keyword (which remained unchanged). This created a weakly constrained sentence frame that generates a less clearly defined context, making the final keywords difficult to predict. To maintain parity of the levels of semantic complexity in the strongly and weakly constrained sentence frames, both frames contained the same number of pointer words. Other changes to the sentence frame were minimized, although in some cases function or filler words were added to or deleted, to maintain naturalness. For some sentences it was not possible to generate a weakly constrained context only by substituting pointer words, so new frames were constructed with the same number of pointer words. For example, “*Meat* from a *cow* is called beef” was difficult to modify by changing only the pointer words, so it became “My *favorite meat* is beef.” Although this meant that some strongly–weakly constrained sentence frame pairs are less similar than other pairs in which only the pointer words differ, the structure and complexity of the sentence frames were kept as similar as possible across pairs.

A second round of cloze tests was conducted, to make sure these sentences were semantically neutral. In this case, a low cloze probability for the final keyword would show that the keyword was now not easy to predict. The upper threshold for inclusion was set at a cloze probability of 40%, in line with previous materials (Block & Baldwin, 2010), and was applied to the most common response given rather than to the only intended keyword.

Cloze test N1 consisted of all 481 neutral sentences and was completed online by nine native (six female, three male) and five proficient non-native (all female, average AoA = 8.60 years) English speakers with an average age of 25.78 years. The native language of the participants in cloze tests N1 and N2 are given in Table 2. The participants had not completed any previous cloze tests and received course credits as compensation. The sentences for which the most common response had a cloze probability under the 40% threshold were retained unmodified (248) or adapted slightly to further weaken their contextual constraint (29; e.g., “My *favorite meat* is beef” became “My *favorite food* is beef”). The nine sentences removed from the predictable condition after cloze test P3 were also removed from the neutral condition at this point, even though they had been under the 40% threshold. The sentences whose most common response had a cloze probability over 40% were adapted to increase their contextual ambiguity (195).

Cloze test N2 included the 224 sentences modified after cloze test N1 and was completed online by 14 native (12 female, two male) and four proficient non-native (three female, one male; average AoA = 9.75 years) English speakers with an average age of 32.61 years. The participants received course credit for their participation and had not completed any of the previous tests. The sentences whose most common response had a cloze probability less than 40% were retained (168 sentences), along with three sentences just over this threshold, but for which the most common response was not the intended keyword. The sentences that still had a cloze probability above the 40% threshold were either removed (15) or modified on the basis of the responses to further weaken their contextual constraint and then were retained (38). This left 457 pairs of predictable and neutral sentences that shared the same final keyword.

### Semantically anomalous sentences

A semantically anomalous counterpart was created for each predictable sentence by substituting the congruous final keyword for one that was incongruous to the highly constrained sentence context. Each incongruous keyword was drawn from the remaining pool of 932 potential keywords and was matched to the corresponding congruous keyword on a number of features: noun type (i.e., a singular countable noun was substituted for another singular countable noun), syllable count, lexical stress pattern, lexical frequency (Brysbaert & New, 2009), phonological neighborhood density (number of words that differ from the target by only one phoneme; Marian, Bartolotti, Chabal, & Shook, 2012), and phonological Levenshtein distance (mean number of changes required to transform the target into its 20 closest phonological neighbors; Balota et al., 2007). The keyword pairs were also matched on concreteness (e.g., “pen” is more concrete than “love”; Wilson, 1988) as far as possible*.* Age of acquisition was not used as a matching criterion, because these data were not available for approximately half of the potential keywords (Wilson, 1988) and because this feature might not be so relevant, since the materials were primarily intended for L2 research. Keyword pairs were also selected to be immediately acoustically distinguishable, with no initial phonological overlap between the two words; initial consonants (singletons or clusters) differed in their place and/or manner of articulation and voicing (e.g., /b/ vs. /s/, /sl/ vs. /tr/), and the first vowel also differed in height and/or roundedness (e.g., /i:/ vs. /o/). None of the incongruous counterparts had been given as responses to the relevant sentence in cloze tests P1–P3. The pool of potential keywords was limited, so it was not possible to find a suitable match for each congruous keyword, meaning that 18 sets of predictable and neutral sentences were removed at this point.

### The complete NNSR sentence set

The development of the NNSR sentences gave a final set of 439 triplets of semantically predictable, neutral, and anomalous sentences that are suitable for use in L2 speech perception research. During the development process, the sentence frames and final keywords used within each triplet were closely matched on a one-to-one basis for a number of factors. To ensure that this had maintained equivalence across the three semantic conditions, the complete sets of sentence frames and final keywords were compared as a whole. Initial comparisons showed that the strongly and weakly constrained sentence frames differed slightly in total word count; the weakly constrained sentence frames were slightly shorter on average than the strongly constrained frames. To correct this, approximately 20 of the shortest weakly constrained sentence frames were lengthened by separating contractions (e.g., *don*’t → *do not*) or adding “filler” words (e.g., *very*, *really*). The adjusted strongly and weakly constrained sentence frame sets then showed very similar mean syllable counts (8.40 vs. 8.35, respectively), total word counts (6.51 vs. 6.39, respectively), and pointer word counts (2.53 vs. 2.52, respectively). Across the strongly and weakly constrained sentence frames, the total numbers of pointer words were equivalent, but the average occurrence of each pointer word across the set was higher for the weakly than for the strongly constrained sentence frames (2.56 vs. 1.79, respectively). This is because the weakly constrained sentence frames tend to use more general pointer words that appear more frequently than the more specific pointer words in the strongly constrained frames. For example, *people* is used a number of times in weakly constrained frames to substitute for more specific pointer words, such as *children*, *students*, and *teachers*, in the strongly constrained frames. More detail about the properties of the strongly and weakly constrained sentence frames is presented in Table 3.

**Table 3:** Properties of the sentence frame sets

	Strongly Constrained	Weakly Constrained
Syllable count	8.40 (1.58)	8.35 (1.32)
Total word count	6.51 (1.15)	6.39 (1.03)
Pointer word count (per sentence)	2.53 (0.49)	2.52 (0.50)
Pointer word count (across whole set)*	1,100 (623 unique)	1,087 (425 unique)
Pointer word frequency (across whole set)	1.79 (1.69)	2.56 (3.57)

Values are given in the form mean (*SD*), except in the case of *

The congruous and incongruous final keywords were also found to be very closely matched on mean syllable count (1.795 vs. 1.797, respectively), lexical frequency (3.14 vs. 3.12, respectively), phonological neighborhood density (12.43 vs. 12.47, respectively), and phonological Levenshtein distance (1.91 vs. 1.89, respectively). However, the congruous keywords showed higher mean concreteness ratings than did the incongruous keywords (543.9 vs. 439.7, respectively). This is because the congruous keywords tend to be more concrete in order to complete the context in semantically predictable sentences, which left a higher proportion of more abstract nouns in the remaining pool of potential incongruous keywords. These properties of the congruous and incongruous keywords are shown in more detail in Table 4.

**Table 4:** Properties of the congruous and incongruous final keyword sets

	Congruous	Incongruous
Syllable count	1.795 (0.841)	1.797 (0.839)
Lexical frequency (SUBTLEX Log10)	3.14 (0.60)	3.12 (0.59)
Phonological neighborhood density (CLEARPOND)	12.43 (13.79)	12.47 (13.83)
Phonological Levenshtein distance (English Lexicon Project)	1.91 (0.87)	1.89 (0.87)
Concreteness (MRC)	543.9 (84.70)	493.7 (105.78)

Values are given in the form mean (*SD*)

Finally, the 439 NNSR sentence triplets were organized into 18 experimental lists of 24 sentences, with the remaining seven triplets forming a training list. The lists were created by distributing the semantically predictable sentences across 18 lists, with a range of keyword syllable counts, sentence syllable counts, final keyword cloze probabilities, and pointer word counts within each list. The lists of semantically neutral and anomalous sentences were created by assigning sentences to the same list as their predictable counterpart in the same triplet. The final sentence triplets are identified via a code comprising the semantic condition (predictable = A, neutral = B, anomalous = C), a list identifier (01–18, plus 00 for the training list), and a sentence identifier (01–24); sentence A0101 is thus Sentence 1 of List 1 for the semantically predictable condition, B0101 is the corresponding semantically neutral sentence, and C0101 is the semantically anomalous sentence.

## Intelligibility testing

### Method

A listening test was conducted in order to assess whether the 18 experimental lists are equivalent in their levels of intelligibility. The NNSR sentences were recorded by four Standard Southern British English (SSBE) talkers (two male, two female). All talkers were native, monolingual English speakers, with an accent typical of southeastern England. Recordings were made digitally in a soundproof recording booth at a sampling rate of 44100 Hz and 24 bits per sample, and the intensity of the recordings was normalized to the same mean intensity after completion. Speech-shaped noise (i.e., white noise filtered to match a talker’s speech spectrum; Van Engen, Phelps, Smiljanic, & Chandrasekaran, 2014) was generated for each talker, based on the averaged long-term spectrum of their recordings.

Since the NNSR sentences are intended for use in L2 speech research, the listening test was conducted with native Spanish-speaking participants in addition to native English-speaking participants. The participants reported no known hearing, language, or learning impairments and grew up speaking only their native language at home. The 16 Spanish-speaking participants (12 female, four male; mean age = 19.38 years, *SD* = 2.02 years, range = 18–24 years) were raised in northeast Spain, and none had ever lived in an English-speaking country. All were first- or second-year students in an English Studies degree at the University of the Basque Country, spoke English at an upper-intermediate or advanced level, and had begun learning English between the ages of 5 and 7. The 16 English-speaking participants (seven female, nine male; mean age = 25.25 years, *SD* = 4.20 years, range = 19–32 years) were all SSBE speakers who grew up in Southern England.

The listening test included only the semantically neutral sentences, because non-native speakers receive less intelligibility benefit from the availability of semantic information when listening to speech in noise than do native speakers (Bradlow & Alexander, 2007). This meant that the English listeners would not gain an extra benefit from the constrained context in the semantically predictable or anomalous sentences, as compared to the Spanish listeners. Testing took place at the University of the Basque Country (Spanish listeners) and at University College London (English listeners). The complete set of 432 neutral sentences was presented over headphones at a comfortable volume. Four noise conditions were presented: embedded in talker-specific speech-shaped noise at + 3-dB, 0-dB, and – 3-dB signal-to-noise ratios (SNRs), and also in quiet. The sentences were presented in a random order and were equally distributed across the combinations of talker and noise conditions. For each participant the sentences appeared in only one of these combinations, but between participants the sentences were counterbalanced so that each sentence was presented at every noise level across the experiment. After each sentence, participants repeated the words they understood, and the experimenter recorded the number of words (pointer words and the final keyword) correctly identified per sentence, to calculate recognition accuracy. Short breaks were given throughout the task.

### Results

**Fig. 1:**
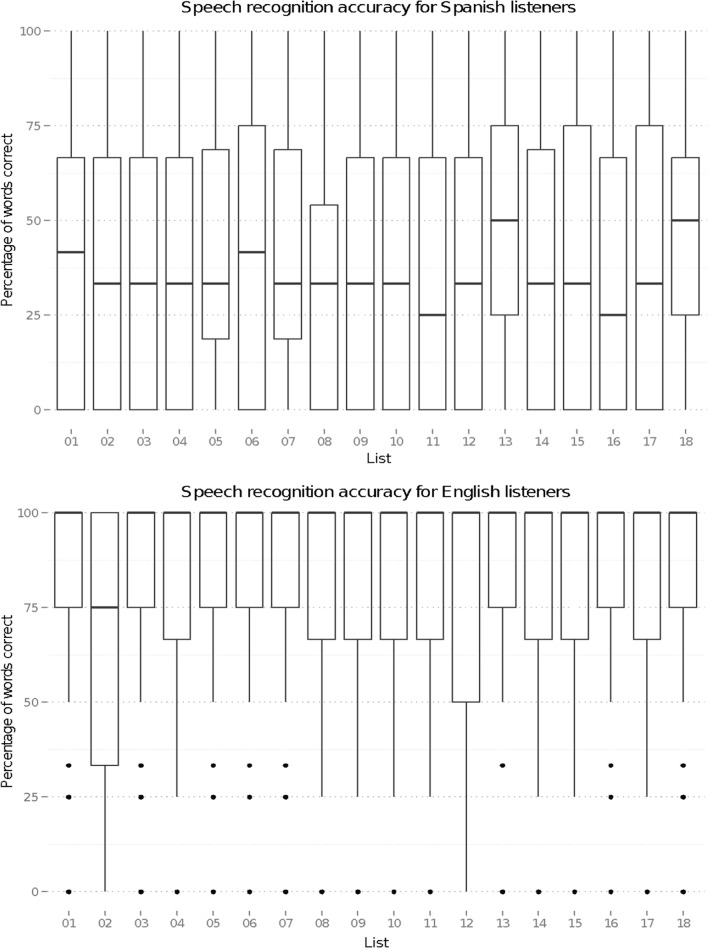
Recognition accuracy of the 18 lists of semantically neutral sentences presented in speech-shaped noise, averaged across the three noise levels

**Fig. 2:**
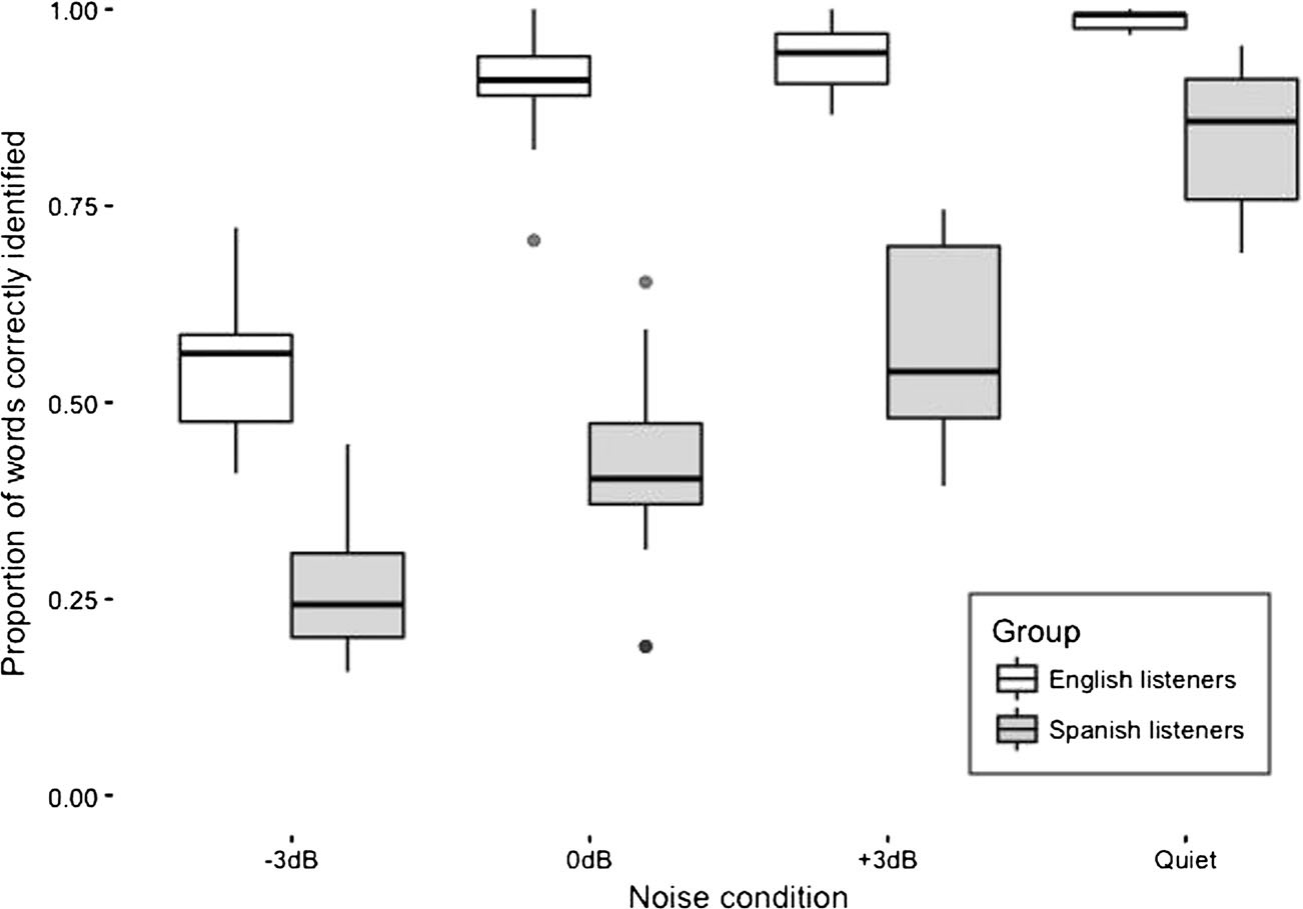
Speech-in-noise recognition accuracy for Spanish and English listeners as a function of noise level

Speech recognition accuracy in noise across the 18 experimental lists for the two listener groups is shown in Fig. 1. This shows recognition accuracy averaged across the three noise levels; the quiet scores were at ceiling, and so have not been included. To investigate any differences among the intelligibility of the lists, separate logistic mixed-effect models were run for each listener group using the glmer function in the lme4 package (Bates, Maechler, Bolker, & Walker, 2014) in the R environment (R Core Team, 2013). The dependent variable was entered as a binomial variable showing recognition accuracy in terms of the ratio of correctly identified words to the total number for each sentence. This was entered into each model, with experimental list and SNR as fixed effects (including the interaction term) and by-participant, by-speaker, and by-sentence random intercepts. No significant effect of list was found on speech recognition accuracy for either listener group: *χ*^2^ = 13.40, *df* = 17, *p* = .709, for Spanish listeners, and *χ*^2^ = 8.14, *df* = 17, *p* = .964, for English listeners. This suggests that the 18 lists of NNSR sentences are of similar intelligibility within each listener group. A significant effect of noise condition was found for both groups: *χ*^2^ = 148.6, *df* = 2, *p* < .001, for Spanish listeners, and *χ*^2^ = 133.8, *df* = 2, *p* < .001, for English listeners. Figure 2 shows that this effect arises as speech recognition accuracy decreases with SNR. No interaction was found between list and noise condition for either group, showing that greater noise levels have similar impacts on the intelligibility of all lists.

This testing suggests that the 18 experimental lists do not differ in intelligibility for L2 and L1 listeners. Only the neutral sentences were tested here, but since the sentences within each triplet are closely matched, it may be expected that the intelligibility of the semantically predictable and anomalous sentences would also be similar across the lists.

## Conclusions and possible applications of the NNSR sentences

The NNSR sentences presented here increase the range of stimulus sets that are available for use in L2 speech perception research. The developmental criteria that informed their design give the material set a range of features that means that the NNSR sentences may be suitable for use in a range of experimental designs, across many aspects of L2 speech research. Some of these potential applications are discussed here.

The most important criterion informing the development of the NNSR was that the set should be suitable for L2 English speakers at the B1 level and above, in addition to more proficient L2 and L1 English speakers. This helps ensure that the NNSR sentences measure speech perception, rather than linguistic knowledge, but also opens up a range of investigations into L2 speech that might not be well served by the materials currently available. For example, the NNSR sentences could be used to track L2 speech acquisition from an early stage in development, either as part of general language tuition, after targeted L2 speech training, or after time in an immersive English environment. The NNSR could also be used to investigate the relative influences of factors such as vocabulary size or phonological memory on speech perception as language proficiency develops.

The second developmental criterion was to produce a large-scale set of sentences; the final NNSR sentence set contains 1,317 sentences across the semantic conditions. For comparison, the BEL sentence set, which is larger than most commonly used materials, contains 500 sentences (Calandruccio & Smiljanic, 2012). The large scale of the NNSR material set means that it provides greater flexibility to allow multiple within-subjects conditions without repeating sentences or limiting conditions to a small number of sentences. This will allow the influence of various factors on L2 speech perception to be investigated in finer detail, by accommodating a greater number of levels within variables (e.g., more SNR levels, speakers, accents, or types of noise masker, or greater amounts of signal degradation) or permitting a greater number of variables, to investigate interactions between factors that might influence L2 speech perception. In addition to allowing more nuanced investigations, the large scale of the NNSR sentences may also mean that fewer participants are required, since there is less need to present stimuli to multiple groups in order to test all conditions, saving time and demand for resources. The NNSR sentences may also be suitable for high-variability phonetic training studies requiring a large number of speakers with little or no repetition of stimuli. However, it should be noted that, because the sentence frames appear in predictable and anomalous conditions and the congruous keywords appear in predictable and neutral conditions, it is not possible to use the full sets of each condition in one experiment without repeating information.

The final developmental criterion was that the NNSR sentences should contain multiple semantic conditions. This was achieved by manipulating the level of semantic context available in the sentences and also the congruity of the final keyword, to create three semantic conditions: predictable, neutral, and anomalous. These multiple conditions increase the range of material sets available for studies investigating the use of semantic cues to support L2 speech perception in unfavorable conditions. Previous investigations in this area have used the SPIN sentences (Shi, 2014) or similar materials adapted for L2 listeners (Bradlow & Alexander, 2007), which both contain high- and low-probability conditions. The BEL sentences have also been used in semantic investigations, but so far only for L1 listeners (Smayda, Van Engen, Maddox, & Chandrasekaran, 2016; Van Engen et al., 2014). The BEL set do not allow for semantic manipulation themselves, so both studies supplemented the BEL sentences with semantically anomalous sentences from the Syntactically Normal Sentence Test (SNST; Nye & Gaitenby, 1974). However, both of these situations are problematic, because the sentences in the two semantic conditions are not matched. In the original and adapted SPIN sentences, high-predictability sentences have a strongly constrained semantic context, but low-predictability sentences are based on a limited variety of extremely simple sentence structures that are essentially meaningless (e.g., “Dad looked at the . . .” and “She talks about the . . .”; Bradlow & Alexander, 2007). The SNST sentences are nonsensical sentences formed of randomly selected words in a rigid structure, such as “The dear neck ran the wife,” and so differ substantially from the BEL sentences. In both cases, the conditions differ not only in the level of semantic context provided, but also in semantic and syntactic complexity, sentence length, and other features that may also influence intelligibility. The carefully matched semantic conditions of the NNSR limit these potential confounds by giving much greater equivalence across factors such as sentence length, keyword frequency, and semantic complexity, in order to minimize the influence of factors other than the availability of contextual information.

The matched semantic conditions also allow the use of the NNSR sentences in electroencephalographic (EEG) investigations of the N400 effect, which is related to semantic processing (Kutas & Hillyard, 1984). In addition to specifically exploring the neural nature of semantic processing, using EEG methods could be useful to explore differences in L2 and L1 speech processing that can be difficult to investigate behaviorally, due to ceiling effects. For example, differences in the N400 effects elicited by speech in quiet for L2 and L1 listeners could be compared to performance in behavioral speech recognition tasks in which differences arise in adverse conditions but might not be seen in quiet. If differences were observed in the N400, this could suggest that qualitative differences in processing in quiet affect word recognition, but that the effect is not so severe as to prevent word recognition. However, equivalent EEG findings could suggest that differences in recognition accuracy arise specifically in relation to the adverse listening condition, and that processing in quiet is more similar. The NNSR set have been used in an investigation of this type, in which the factors affecting processing of accented speech by L2 and L1 listeners were explored using behavioral and EEG methods (Stringer & Iverson, in press).

In conclusion, the NNSR sentences presented in this article are a new material set that increases the range of L2-specific materials available for speech research. They are linguistically accessible to a wider range of L2 speakers of English than are many existing material sets, but they are also of a sufficient complexity to allow administration to more proficient L2 and L1 English speakers. The design of the NNSR set also allows flexibility in experimental design, because multiple semantic conditions and the large scale of the set mean that the NNSR sentences can be used in a variety of experimental designs and also allow multiple within-subjects conditions without repeating items. The full stimulus set (with sentence properties) is available through this link: http://bit.ly/nnsrsent.
